# Selective Pharmacological Inhibition of NOX2 by GSK2795039 Improves Bladder Dysfunction in Cyclophosphamide-Induced Cystitis in Mice

**DOI:** 10.3390/antiox12010092

**Published:** 2022-12-30

**Authors:** Mariana G. de Oliveira, Fabíola Z. Monica, Gabriela R. Passos, Jamaira A. Victorio, Ana Paula Davel, Anna Lethicia Lima Oliveira, Carlos A. Parada, Carlos A. L. D’Ancona, Warren G. Hill, Edson Antunes

**Affiliations:** 1 Department of Translational Medicine, Pharmacology Area, Faculty of Medical Sciences, University of Campinas (UNICAMP), Alexander Fleming St., Campinas 13083-881, SP, Brazil; 2 Department of Structural and Functional Biology, Institute of Biology, University of Campinas, Campinas 13083-881, SP, Brazil; 3 Laboratory of the Study of Pain, Department of Structural and Functional Biology, Institute of Biology, University of Campinas, Campinas 13083-881, SP, Brazil; 4 Department of Surgery, Division of Urology, Faculty of Medical Sciences, University of Campinas, Campinas 13083-881, SP, Brazil; 5 Laboratory of Voiding Dysfunction, Nephrology Division, Department of Medicine, Beth Israel Deaconess Medical Center, Harvard Medical School, Boston, MA 02115, USA

**Keywords:** cystometry, urothelium, void spot assay, setanaxib

## Abstract

Interstitial cystitis/bladder pain syndrome (IC/BPS) is a chronic inflammatory disease without consistently effective treatment. Among the many mediators implicated in cystitis, the overproduction of reactive oxygen species (ROS) seems to play a key role, although the main source of ROS remains unclear. This study aimed to investigate the contribution of NADPH oxidase (NOX) isoforms in ROS generation and the voiding dysfunction of cyclophosphamide (CYP, 300 mg/Kg, ip, 24 h)-induced cystitis in adult female mice, a well-recognized animal model to study IC/BPS, by using GKT137831 (5 mg/Kg, ip, three times in a 24 h period) or GSK2795039 (5 mg/Kg, ip, three times in a 24 h period) to inhibit NOX1/4 or NOX2, respectively. Our results showed that treatment with GSK2795039 improved the dysfunctional voiding behavior induced by CYP, reduced bladder edema and inflammation, and preserved the urothelial barrier integrity and tight junction occludin expression, besides inhibiting the characteristic vesical pain and bladder superoxide anion generation. In contrast, the NOX1/4 inhibitor GKT137831 had no significant protective effects. Taken together, our in vivo and ex vivo data demonstrate that NOX2 is possibly the main source of ROS observed in cystitis-induced CYP in mice. Therefore, selective inhibition of NOX2 by GSK2795039 may be a promising target for future therapies for IC/BPS.

## 1. Introduction

Interstitial cystitis/bladder pain syndrome (IC/BPS) is a chronic and debilitating inflammatory condition that predominantly affects women and is characterized by suprapubic pain related to bladder filling, accompanied by other symptoms, such as increased frequency, in the absence of urinary infection or other obvious pathology [1]. Interstitial cystitis remains a challenge in healthcare, given the unclear and multifactorial aetiology and the lack of effective therapies, with most of them having a low degree of evidence [2]. Cyclophosphamide (CYP) is an alkylating agent indicated to treat many forms of cancer and autoimmune diseases, but hemorrhagic cystitis is a frequent side effect of CYP, occurring in 10 to 40% of patients receiving CYP due to the renal excretion of drug metabolites, particularly acrolein [3]. Additionally, CYP is commonly used as an experimental model for IC/BPS in rodents [4]. Mice exposed to CYP undergo dramatic voiding changes toward a dysfunctional hyperactive phenotype, as characterized by increased voiding frequency and small urinary volumes, associated with severe inflammation, pain, and bladder hypocontractility in vitro [4,5,6,7]. Among the many mediators implicated in IC/BPS pathophysiology, the overproduction of reactive oxygen species (ROS) seems to play a key role [7,8,9,10,11]. In the bladders of CYP-injected mice, we have previously shown a significant increase in superoxide anion (O_2_^−^) generation, mainly in the urothelial layer, the prevention of which (by the soluble guanylyl cyclase activator BAY 58-2667) attenuated the bladder dysfunction [5]. Recently, increased levels of urinary oxidative species were reported in samples from IC/BPS patients, and these have been proposed as novel biomarkers for this disease [12].

Antioxidants and ROS scavengers are a common strategy to overcome oxidative stress bladder injury and these drugs exhibit great therapeutic potential in preclinical studies [7,8,13,14]. On the other hand, clinical trials with IC/BPS patients are scarce and only limited evidence is available [15,16]. Additionally, ROS are now recognized as modulators of numerous cell signaling pathways by redox-based mechanisms, far from the traditional viewpoint of their role as damaging molecules [17], implying that non-specific ROS elimination may counteract its beneficial physiological effects. Therefore, therapies selectively targeting dysregulated ROS generators are actively being sought; however, no study has elucidated the source of the exaggerated ROS in CYP-induced cystitis.

Major sources of ROS production are the mitochondrial oxidases, uncoupled endothelial nitric oxide synthase (eNOS) and NAPDH oxidases (NOX) family [18]. The NOX family has gained prominence as they produce ROS, O_2_^−^, or H_2_O_2_ as their primary and sole function [19]. Seven members of the NOX family (NOX1, NOX2 [*aka* gp91^phox^], NOX3, NOX4, NOX5, DUOX1, and DUOX2) have been identified and their specific structure, distribution, and mechanism of activation have been extensively investigated (for a comprehensive review, see [20]). In the lower urinary tract, both NOX2 and NOX4 are expressed in the mouse urethra and prostate [21,22] and in the human bladder [23,24], but the exact role of NOX in bladder pathophysiology remains poorly explored. Since excessive NOX-derived ROS production is a common feature of several diseases [19], NOX members represent pharmacological targets of great interest. Ideally, a NOX inhibitor should not have intrinsic antioxidant activity or inhibit other sources of ROS, otherwise its effects might be ineffective or even deleterious [25]. The high homology among the different NOX isoforms is critical for the development of these compounds [20]. Several reportedly isoform-selective NOX inhibitors have been recently developed [25], including GKT137831 (Setanaxib), which exhibits preferential inhibition of NOX1 and NOX4, and GSK2795039, a competitive NOX2 inhibitor. Setanaxib is the first-in-class drug currently under phase II clinical trials for idiopathic pulmonary fibrosis and diabetic kidney disease. Therefore, we hypothesized that increased ROS generation as a result of NOX isoform(s’) overactivation is essential for CYP-induced bladder cystitis and that NOX inhibitors could attenuate CYP-induced bladder functional and molecular alterations. We therefore designed the present study to evaluate whether NOX1/4 or NOX2 inhibition (by using GKT137831 or GSK2795039, respectively) improves ROS-generation, bladder hyperactivity, and inflammatory and nociceptive responses related to CYP-induced cystitis in mice.

## 2. Materials and Methods

### 2.1. Chemicals

GKT137839 was obtained from Cayman Chemicals (Ann Arbor, MI, USA); GSK2795039 was from MedChemExpress (Monmouth Junction, NJ, USA) and all other chemicals were from Sigma (St. Louis, MO, USA), unless otherwise specified.

### 2.2. Animals

Female C57BL/6, 12 weeks old, were housed in cages (*n* = three per cage) located in ventilated cage shelters with constant humidity of 55 ± 5% and temperature of 24 ± 1 °C under a 12 h light–dark cycle and received standard food ad libitum. Euthanasia was performed by isoflurane overdose, in which the animals were exposed to a concentration greater than 5% until one minute after breathing stopped. Cervical dislocation was performed to confirm the euthanasia. After euthanasia, the total body and bladder weights, as well as the relative bladder weight (bladder to body ratio), were determined. The animal studies are reported in compliance with the ARRIVE guidelines.

### 2.3. Experimental Design

The study was divided into two parts, the first of which consisted of a time-course evaluation of CYP-induced effects and oxidative stress over time. Specifically, the mice were randomly divided into five subgroups (*n* = six mice each), representing the control group (saline injection) and the time-points 3, 6, 12, and 24 h after CYP injection (300 mg/Kg, i.p., in saline) groups. All of the animals were euthanized at the same time to avoid circadian fluctuations.

The second part was carried out to verify whether the NOX inhibitors GKT137831 and GSK2795039 attenuate CYP-induced cystitis. The time-point of 24 h after CYP injection (24 h) was selected. Briefly, the animals were randomly allocated to four sub groups (n = six/group), namely (i) control group, injected with saline (10 mL/Kg) plus three sequential injections of vehicle (details below); (ii) CYP, injected with CYP (300 mg/Kg, ip, in saline) plus three injections of vehicle; (iii) CYP + GSK2795039, injected with CYP (300 mg/Kg, ip, in saline) plus three injections of GSK2795039 (5 mg/Kg, ip, in vehicle); and (iv) CYP + GKT137831 injected with CYP (300 mg/Kg, ip, in saline) plus three injections of GKT137831 (5 mg/Kg, ip, in vehicle). Injections of GSK2795039, GKT137831 or vehicle started 1 h after CYP injection to avoid interferences with CYP absorption and were repeated at approximately 8 h intervals. The vehicle for both GSK2795039 and GKT137831 was Cremophor^®^ 15% in saline, and the optimal dose regimen were estimated based on previous studies [26,27]. Pharmacokinetic characterization of GSK2795039 in C57BL/6 mice revealed poor bioavailability and high clearance, resulting in an elimination half-life of > 2 h in mice and rats [26], while GKT137831 has a better pharmacokinetic profile with one or two daily doses [28].

### 2.4. Measurement of Superoxide Dismutase (SOD) Activity in Bladder Tissue

Bladder tissue was homogenized in ice-cold PBS containing protease inhibitors. The homogenates were centrifuged, and the resulting supernatants were used for the assay. The measurement of total SOD activity was determined using an enzymatic assay kit (Cayman Chemical, Catalog No 706002, Ann Arbor, MI, USA), according to the manufacturer’s protocol. Absorbance was monitored at 440 nm. Data were normalized to mg of protein.

### 2.5. Measurement of Peroxidase Activity and H_2_O_2_ Levels

Bladder tissue was homogenized in ice-cold PBS containing protease inhibitors. The homogenates were centrifuged, and the resulting supernatants were used for the assay. The H_2_O_2_ levels in the homogenates were measured using a hydrogen peroxide assay kit (Cell BioLabs, San Diego, CA, USA), according to the manufacturer’s protocol. The absorbance was monitored at 540 nm, and the H_2_O_2_ concentrations were determined using the standard curve. Data were normalized to mg of protein.

### 2.6. Measurement of Superoxide Anion (O_2_^−^)

The oxidative fluorescent dye dihydroethidium (DHE) was used to evaluate in situ ROS generation. The bladders were embedded in a freezing medium and transverse sections (12 µm) were obtained on a cryostat, collected on glass slides, and equilibrated for 10 min in Hank’s solution (1.6 mM CaCl_2_, 1.0 mM MgSO_4_, 145.0 mM NaCl, 5.0 KCl, 0.5 mM NaH_2_PO_4_, 10.0 mM Glucose, and 10.0 HEPES, pH 7.4). Fresh Hank’s DHE solution (2 µM) was applied to each tissue section, and the slides were incubated in a light-protected humidified chamber at 37 °C for 30 min. In some experiments, the non-selective nitric oxide synthase inhibitor L-NAME (1 mM) was applied over the tissue slice 30 min before DHE incubation. Images were obtained with a microscope (Eclipse 80i, Nikon, Tokyo, Japan) equipped for epifluorescence (excitation at 488 nm and, emission at 610 nm) and a digital camera (DS-U3, Nikon). The fluorescence was detected with a 585 nm long pass filter. The number of nuclei labeled with ethidium bromide in the detrusor smooth muscle and urothelium wall was automatically counted using Image J software (NIH, Bethesda, MD, USA) and expressed as labeled nuclei per millimeter squared.

### 2.7. qPCR—RNA Extraction

Total RNA was extracted from freshly dissected bladders using TRIzol^®^ reagent (Invitrogen, Hattiesburg, MS, USA) according to the manufacturer’s protocol. The DNase-treated RNA samples were then transcribed with the High-Capacity Reverse Transcription Kit^®^ (Applied Biosystems, Foster City, CA, USA). cDNA concentrations were quantified using a spectrophotometer (Nanodrop Lite^®^, Thermo Scientific, Waltham, MA, USA).

### 2.8. qPCR—Reverse Transcription

Synthetic oligonucleotide primers (Table 1) were obtained from Integrated DNA Technologies (Coralville, IA, USA). The reactions were performed with 10 ng cDNA, 6 µL SYBR Green Master Mix^®^ (Life Technologies, Carlsbad, CA, USA), and the optimal primer concentration, in a total volume of 12 µl. Real-time PCR was performed in the equipment StepOne-Plus^®^ Real Time PCR System (Applied Biosystems, Waltham, MA, USA). The reaction program was 95 °C for 10 min, followed by 40 cycles of 95 °C for 15 s then 60 °C for 1 min. At the end of a normal amplification, a degradation time was added, during which the temperature increased gradually from 60 °C to 95 °C. The threshold cycle (Ct) was defined as the point at which the fluorescence rises appreciably above the background fluorescence. Two replicas were run on the plate for each sample, and each sample was run twice independently. All of the experiments included melt curves to monitor reaction integrity. The 2^−ΔΔCt^ method was utilized to analyze the results, which were expressed by the difference between the Ct values of the chosen genes and the average of housekeeping genes β–actin and 18s ribosomal subunit.

### 2.9. Western Blotting

Western blot analysis to investigate eNOS dimerization was performed as previously described [29]. Total protein extracts were obtained from homogenized bladders in cold lysis buffer containing Tris-HCl (50 mM), NaCl (150 mM), ethylenediaminetetraacetic acid (EDTA, 0.5 mM), phenylmethylsulfonyl fluoride (PMSF, 1 mM), Na_3_VO_4_ (1 mM), 0.2% Nonidet P-40, dithiothreitol (0.1 mM), and a protease inhibitor cocktail (2 μL/mL) for 1 h at 4 °C. Non-boiled samples (50 μg) and boiled (5 min, 95 °C) control samples were separated by 6% sodium dodecyl sulfate–polyacrylamide gel electrophoresis (SDS-PAGE) at 4 °C for eNOS dimer analysis. Then, the proteins were transferred to polyvinylidene fluoride (PVDF) membranes (GE Healthcare, Little Chalfont, BUX, UK) overnight at 4 °C, blocked for 90 min at room temperature with 5% albumin in Tris-buffer (Tris 10 mM, NaCl 100 mM and Tween 20 0.1%), then incubated overnight at 4 °C with anti-eNOS (1:1000; #610297 BD Transduction, Franklin Lakes, NJ, USA). Protein expression was detected using a specific, horseradish peroxidase-conjugated secondary antibody (1:5000; #7076S Cell Signaling, Danvers, MA, USA) and enhanced chemiluminescence (ECL) solution. Densitometry was carried out with ImagLab (Biorad, Hercules, CA, USA). The ratio between the eNOS homodimers (200–260 kDa) and monomers (~135 kDa) was also determined.

### 2.10. Bladder Histology

The bladders were removed, fixed with 10% phosphate-buffered formalin for 24 h, dehydrated in ethanol, and embedded in paraffin. The tissues were sliced (5-μm sections) on a microtome (Leica, Wetzlar, Germany), dewaxed in xylene, rehydrated in gradient alcohol, and stained with hematoxylin–eosin for light microscopy examination using 5× and 20× objectives. Digital images were obtained with a microscope Eclipse 80i (Nikon, Tokyo, JP) equipped with a digital camera (DS-U3, Nikon). The thickness of the urothelium and detrusor smooth muscle was evaluated using the ImageJ Software (Version 1.46r), according to a previous study [30].

### 2.11. Void Spot Assay (VSA)

The VSAs were performed as described [31,32] during the last 4 h of CYP exposure. Mice were moved individually to empty mouse cages with precut qualitative filter paper (250 g) on the bottom. They were provided with food, but no water. After 4 h, the filter papers were removed and were allowed to dry before being photographed under UV light (365 nm). During image analysis, any overlapping spots were outlined with the drawing tool in Image J, copied, and moved to an empty area of the filter. By using a machine learning algorithm developed by one of us (W.G.H) who was trained on 60 void spot filter images, we have quantified the number of void spots, the number of primary voids (PV; >20 μL), the total urine volume, the mean PV volume, and the number of microvoids (<20 μL). This assay was performed on three consecutive days before any injections to habituate the animals, then from the 20th to 24th hour after saline or CYP injection, at always around the same hours during daytime and in a quiet room.

### 2.12. Anesthetized Cystometry

The mice were anesthetized by intraperitoneal injection of urethane (1.0 g/Kg). A 1 cm abdominal incision was made to expose the bladder and a 25 g cannula was inserted into the bladder dome. The cannula was connected to a three-way tap, of which one port was connected to the infusion pump through a PE-50 catheter. Before starting cystometry, the bladder was emptied and continuous cystometry was performed by infusing saline into the bladder at 0.6 mL/h for 45 min after the end of the first micturition cycle. The following parameters were assessed: baseline pressure (minimum pressure between two voids), capacity (volume needed to induce first void), threshold pressure (pressure immediately before a void), compliance (ratio between capacity and threshold pressure), peak pressure (pressure reached during voiding), and voiding frequency [6]. One mouse was used for each cystometrogram and euthanatized immediately after the experimental protocol.

### 2.13. Mechanical Allodynia Assessment by von Frey

To quantify and analyze pain levels, the mechanical nociceptive threshold was measured through an electronic von Frey apparatus (Insight, Sao Paulo, Brazil) adapted for mice [33]. Each mouse was placed in a plexiglass chamber measuring 6 cm × 6 cm × 12 cm with the floor consisting of a perforated metal grid (1 cm × 1 cm) to allow for the pain test to be administered. Each mouse was placed in its individual chamber and allowed to acclimate to its surroundings for at least 60 min before stimulation. The test consisted in stimulating the pelvic region with a hand-held force transducer adapted with a polypropylene tip. A gradual increase in pressure to the supra-pubic region was applied and the stimulus was automatically discontinued, and its intensity was recorded when sharp retraction of the abdomen, immediate licking in the region of stimulation, and jumping were observed. The tester was blinded to the treatment each group received. Pain assays were performed prior to any injections (baseline), 4 h after CYP or saline injections, and prior to animal sacrifice/tissue harvesting. Data are expressed as the mechanical nociceptive threshold (g).

### 2.14. Statistical Analysis

Data are expressed as the mean ± standard error of the mean (SEM) of six animals per group. The group sizes referred to independent values not replicates. A Shapiro–Wilk test was performed to test normal distribution. The software GraphPad Prism Version 6 (GraphPad Software Inc., La Jolla, CA, USA) was used for all of the statistical analysis. All statistical comparisons were pre-planned and reported irrespective of outcome, whether *p* was <0.05 or not. Comparisons among three groups were evaluated using one-way (in the case of one variable) or two-way (in the case of two or more variables) analysis of variance (ANOVA), followed by Tukey’s post-hoc test. *p* < 0.05 was taken as showing a significant difference.

## 3. Results

### 3.1. Time Course of ROS Generation and Degradation in CYP-Induced Cystitis

**Figure 1:**
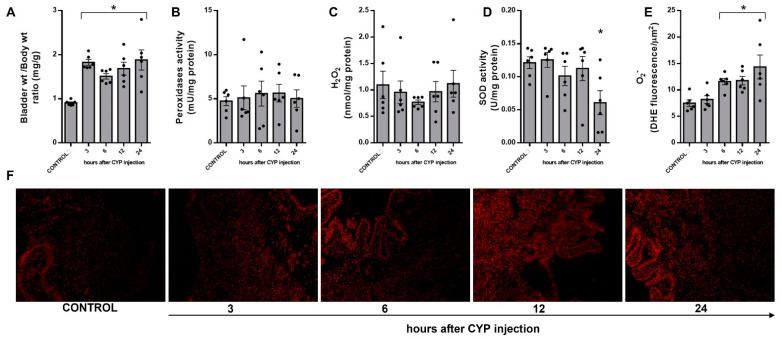
Temporal changes in the female mouse urinary bladder after cyclophosphamide (CYP; 300 mg/Kg, ip) injection in relation to the control group (saline). (**A**) Bladder weight/total body weight ratio. Changes in peroxidases (**B**), hydrogen peroxide (H_2_O_2_; (**C**)) levels, and superoxide dismutase (SOD; (**D**)) activity were measured by colorimetric assays. Superoxide anion (O_2_^−^) levels were determined by a dihydroethidium (DHE) fluorescence assay (**E**,**F**). The fluorescence intensity was quantified using ImageJ. Summary data (mean ± SEM) represent *n* = 6 for each group/time point. Significance was tested by one-way ANOVA followed by multiple comparisons by Tukey’s post-hoc test. * *p* < 0.05 vs. the CONTROL.

Significant increases in the bladder weight/total body weight ratio (Figure 1A) were observed after CYP exposure at all time points evaluated in relation to the control group. We next evaluated peroxidases and superoxide dismutase (SOD) activities in the bladder tissues, as these enzymes form the front line of defense against oxidative stress. The activity of peroxidases (Figure 1B) and H_2_O_2_ levels (Figure 1C) followed a linear rate and did not significantly change after CYP exposure. SOD activity was maintained similar to the control group during 3 to 12 h of CYP exposure, reducing by about 50% after 24 h (*p* < 0.05, Figure 1D). Moreover, O_2_^−^ levels, measured by DHE staining, increased progressively from 6 to 24 h in both smooth muscle and urothelial layers (*p* < 0.05, Figure 1E,F).

### 3.2. Coupled eNOS Is Preserved, but NOX Isoforms Are Modulated in CYP-Induced Cystitis

**Figure 2:**
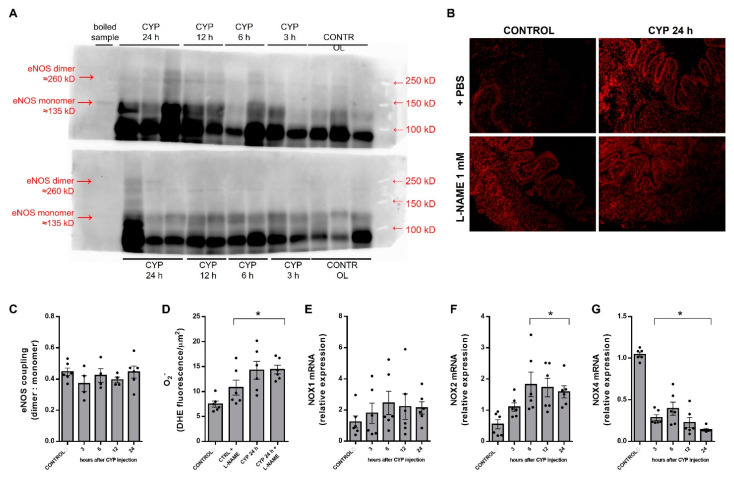
Temporal changes in endothelial nitric oxide synthase (eNOS) expression (**A**) and functional eNOS coupling (**B**) in bladder lysates from the control (saline) and cyclophosphamide (CYP; 300 mg/Kg, ip)-injected animals. The bladder samples were processed in non-denaturing conditions (low temperature electrophoresis) to analyze structural coupling (dimers to monomers ratio; (**C**)). A boiled sample was used as a monomer control. The densitometry was calculated by ImageLab (Biorad, Hercules, CA, USA). A dihydroethidium (DHE) assay was performed in the bladders from CTRL and the 24 h CYP-injected animals, pre-incubated or not with non-selective eNOS inhibitor L-NAME (1 mM, 30 min). The fluorescence intensity was quantified using ImageJ (**D**). The temporal changes in mRNA expression levels of NOX1 (**E**), NOX2 (**F**), and NOX4 (**G**) were evaluated by RT-PCR and expressed as relative to the housekeeping genes. Summary data (mean ± SEM) represent *n* = 4–6 for each group/time point. Significance was tested by one-way ANOVA followed by multiple comparisons by Tukey’s post-hoc test. * *p* < 0.05 vs. CONTROL.

To determine the main source of the excessive O_2_^−^ production we evaluated eNOS coupling and NOX mRNA expression levels. Since uncoupling of eNOS increases O_2_^−^ levels and is detrimental to NO, we evaluated both structural (Figure 2A) and functional (Figure 2B) eNOS coupling in the bladders from control and CYP-exposed animals. Structurally, the dimer to monomers expression ratio (Figure 2C) remained similar to the control in CYP-exposed animals over time. We further examined the effect of CYP in eNOS functional uncoupling by measuring the O_2_^−^ levels in the bladder tissues incubated with the NO synthesis inhibitor L-NAME (1 mM, 30 min; Figure 2B,D). In the control animals, L-NAME by itself significantly increased the O_2_^−^ levels. In CYP-exposed animals, however, inhibition of eNOS with L-NAME did not affect O_2_^−^ formation, thus discarding eNOS as a potential O_2_^−^ source in this model.

mRNA levels of NOX isoforms were measured in whole bladder tissues in the control and CYP groups (Figure 2E–G). In the control samples, NOX2 (Ct: 27.9 ± 0.5) and NOX4 (Ct: 28.9 ± 1.4) were more expressed than NOX1 (Ct: 32.0 ± 0.7). In the CYP group, mRNA expression for NOX2 (Figure 2F) increased progressively, peaked at 6 h, and plateaued for 24 h (*p* < 0.05). In contrast, NOX4 mRNA (Figure 2G) decreased rapidly by more than 80% in relation to the control at all of the time points evaluated (*p* < 0.05), whereas NOX1 did not change after CYP exposure at any time point (Figure 2E). We have not determined the protein expression for NOX2 or NOX4 isoforms because of a lack of available isoform-specific antibodies. To further explore the role of NOX2 and NOX4, we selected the 24 h CYP exposure time and treated the animals with GKT137831 or GSK2795039 during this period.

### 3.3. Inhibition of NOX2, but Not NOX1/4, Attenuates CYP-Induced Bladder Histological Damage

**Figure 3:**
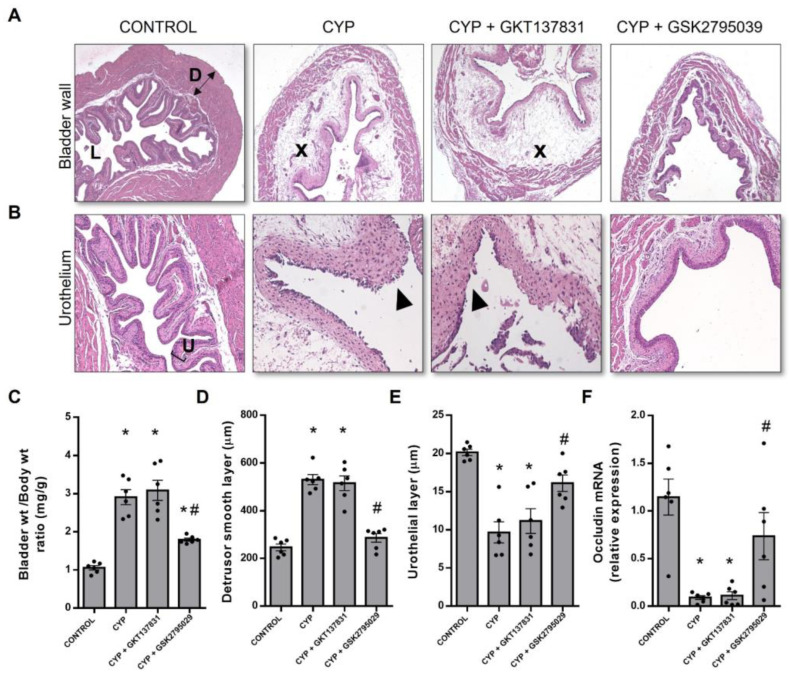
Histological examination of hematoxylin–eosin-stained bladder sections of the control (saline) and cyclophosphamide (CYP, 300 mg/Kg, ip, 24 h)-injected animals untreated or treated (three doses in 24 h) with the NOX1/4 inhibitor GKT137831 (5 mg/Kg, ip) or NOX2 inhibitor GSK2795039 (5 mg/Kg, ip). The upper panel shows the bladder wall ((**A**), 5× magnification) while the lower panel highlights the urothelium ((**B**), 20× magnification). The bladder weight/total body weight ratio (**C**) were determined. The thickness of the detrusor smooth muscle and urothelial layers are shown in (**D**,**E**), respectively. The mRNA expression levels of occludin (**F**) were determined by RT-PCR and expressed as relative to the housekeeping genes. Summary data (mean ± SEM) represent *n* = 6 for all of the groups. Significance was tested by two-way ANOVA followed by multiple comparisons by Tukey’s post-hoc test. * *p* < 0.05 vs. CONTROL; # *p* < 0.05 vs. CYP. D, detrusor smooth muscle; L, lumen; U, urothelium. The ‘x’ highlights severe edema and the arrowhead highlights urothelial denudation.

Histopathological evaluation of the bladder showed alterations after CYP injection (Figure 3A,B) characterized by submucosal edema. This was also evidenced by the increase in bladder weight/body weight ratio (Figure 3C, *p* < 0.05 vs. the control), and muscularis edema and disorganization, which leads to an increase in the detrusor smooth muscle layer (Figure 3D; *p* < 0.05 vs. the control). Mucosal abrasion evidenced by thinning of the urothelial layer was also observed in the CYP group (Figure 3E, *p* < 0.05 vs. the control). Treatment with GKT137831 did not attenuate any of these CYP-induced changes (Figure 3A–E). In contrast, GSK2795039 treatment was highly effective in attenuating CYP-induced cystitis, as demonstrated by the significant reductions in the submucosal and muscularis edema (Figure 3A) in CYP + GSK2795039 in relation to CYP (Figure 3A), even though the bladder weight ratio was still higher than the controls (*p* < 0.05, Figure 3C). More importantly, the urothelium of the CYP + GSK2795039-treated animals remained almost intact (Figure 3B,E), although a few areas of urothelial abrasion are revealed. Additionally, CYP drastically reduced the mRNA expression of the tight junction protein occludin (Figure 3F, *p* < 0.05 vs. the control), which was unaffected by GKT137831 treatment, but markedly attenuated by GSK279539 (*p* < 0.05 vs. CYP).

### 3.4. Inhibition of NOX2, but Not NOX1/4, Improved CYP-Induced Bladder Dysfunction In Vivo

**Figure 4:**
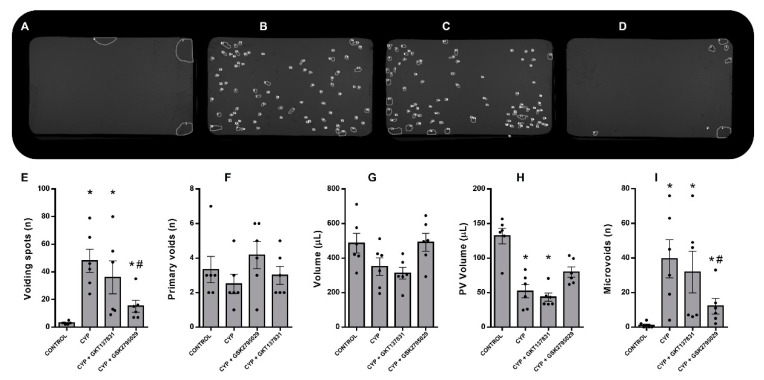
Void spot filter papers imaged under UV light (**A**–**D**). Automated spot detection of the control (saline) and cyclophosphamide (CYP, 300 mg/Kg, ip, 24 h)-injected animals untreated or treated (three doses in 24 h) with the NOX1/4 inhibitor GKT137831 (5 mg/Kg, ip) or NOX2 inhibitor GSK2795039 (5 mg/Kg, ip). The number of voiding spots (**E**), the number of primary voids (PV; greater than 20 μL; (**F**)), the total volume (**G**), the PV volume (**H**), and the number of microvoids ((**I**); less than 20 μL and greater than 2 μL). Summary data (mean ± SEM) represent *n* = 6 for all of the groups. Significance was tested by two-way ANOVA followed by multiple comparisons by Tukey’s post-hoc test. * *p* < 0.05 vs. CONTROL; # *p* < 0.05 vs. CYP.

We initially studied the effects of NOX inhibitors in freely moving mice by the VSA method. Figure 4 A–D shows examples of filter papers of mice from four groups, following 4 h of conscious behavior and voluntary voiding. Summarized data are shown in Figure 4E–I. Control animals (Figure 4A) exhibited only a few urinary spots, at the corners of the paper, and with a large volume. As previously described, mice injected with CYP (Figure 4B) exhibited a dramatic increase in the number of urinary spots (Figure 4E), distributed all over the paper, indicating a hyperactive voiding profile. The number of PV (Figure 4F) and total voided volume (Figure 4G) were similar between the CYP and controls groups. However, the volume of the PV (Figure 4H) was significantly reduced, whereas the number of microvoids was increased in CYP-injected mice (*p* < 0.05 vs. the control). Treatment with GKT137831 (Figure 4C) had no effect on any of these CYP-induced alterations. More interestingly, GSK2795039 significantly attenuated all CYP-induced voiding alterations (Figure 4D), as the animals’ voiding behavior was similar to the controls in the corner zones of the cage, although the number of total and microvoids were still increased compared to the control groups (*p* < 0.05).

**Figure 5:**
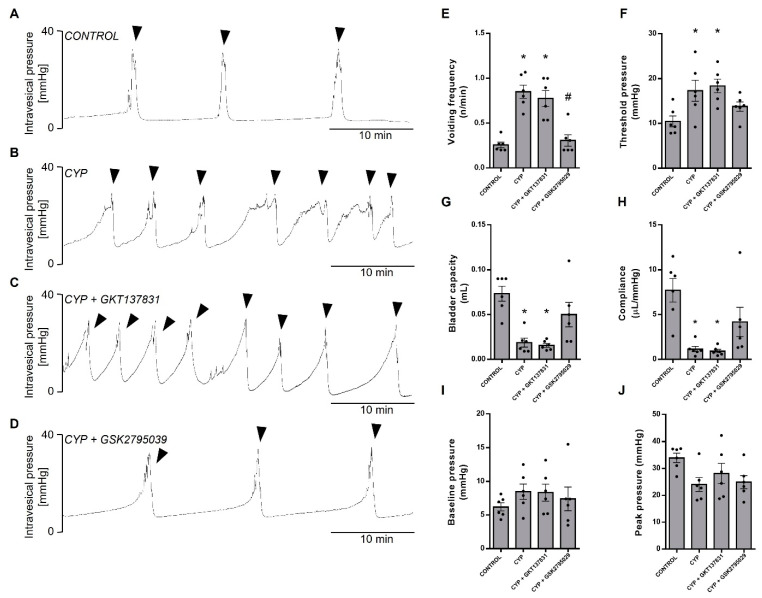
Cystometry under urethane anesthesia and continuous saline filling (0.6 mL/h). Representative cystometric tracings (**A**–**D**) of the control (saline) and cyclophosphamide (CYP, 300 mg/Kg, ip, 24 h)-injected animals untreated or treated (three doses in 24 h) with the NOX1/4 inhibitor GKT137831 (5 mg/Kg, ip) or NOX2 inhibitor GSK2795039 (5 mg/Kg, ip). Changes in the cystometric parameters (**E**) basal pressure, (**F**) threshold pressure to initiate voiding, (**G**) bladder capacity, (**H**) compliance during the filling phase, (**I**) peak pressure, and (**J**) voiding frequency. Summary data (mean ± SEM) represent *n* = 6 for all of the groups. Significance was tested by two-way ANOVA followed by multiple comparisons by Tukey’s post-hoc test. * *p* < 0.05 vs. CONTROL; # *p* < 0.05 vs. CYP. Arrowheads indicates voidings. One mouse was used for each cystometrogram.

We next examined the urodynamic characteristics by cystometry under animal anesthesia. Figure 5A–D shows representative cystometry tracings of mice from four groups, showing the intravesical pressure changes following 45 min of continuous filling (0.6 mL/h). In relation to the controls (Figure 5A), CYP-injected mice (Figure 5B) displayed irregular micturition patterns characterized by significant increases in voiding frequency (Figure 5E), as the intervals between voiding shortened, and threshold pressure (Figure 5F), as the intravesical pressures increased proportionately with filling rate, both of which were accompanied by reductions in bladder capacity (Figure 5G) and compliance (Figure 5H). No changes were observed for baseline (Figure 5I) or peak pressures (Figure 5J). Similarly to the VSA tests, treatment with GKT137831 (Figure 5C) had no effect in any of these CYP-induced urodynamic alterations, but GSK2795039 significantly attenuated all CYP-induced voiding alterations (Figure 5D).

### 3.5. Pain Induced by CYP Was Fully Inhibited by GSK2795039, but Not by GKT137831

**Figure 6:**
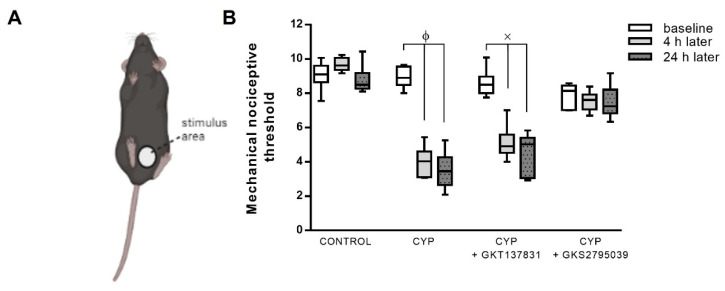
Nociceptive response to mechanical stimulation of the suprapubic region (**A**), an indicator of pain referred from the bladder, of the control (saline) and cyclophosphamide (CYP, 300 mg/Kg, ip, 24 h)-injected animals untreated or treated (three doses in 24 h) with the NOX1/4 inhibitor GKT137831 (5 mg/Kg, ip) or NOX2 inhibitor GSK2795039 (5 mg/Kg, ip). The responses (**B**) were quantified at the baseline (before any injections; white boxes), 4 h after saline or CYP injection (gray boxes), and 24 h after saline or CYP injection (gray dotted boxes). Summary data (mean ± SEM) represent *n* = 6 for all of the groups. Significance was tested by two-way ANOVA followed by multiple comparisons by Tukey’s post-hoc test. ϕ *p* < 0.05 vs. CYP-baseline; × *p* < 0.05 vs. CYP + GKT147831 baseline.

Nociceptive response was evaluated by von Frey testing in the suprapubic area (Figure 6A,B). No significant differences were identified at the baseline, before any injections, between the groups. The control animals exhibited a similar behavior at the time points evaluated. However, a reduced nociceptive threshold was observed as early as 4 h after CYP exposure and was maintained lower at 24 h when compared with the respective baseline. Pelvic hypersensitivity was not ameliorated by GKT137831 treatment but was significantly inhibited by GSK2795039 treatment, achieving a response similar to the control animals.

### 3.6. NOX2-Derived Superoxide Anion Generation

**Figure 7:**
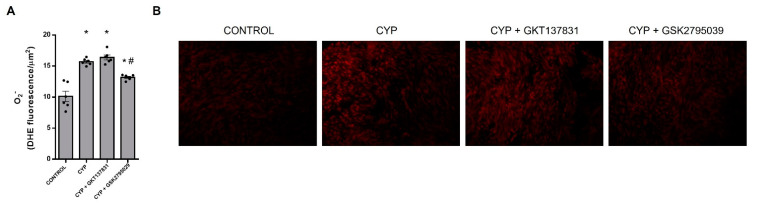
Superoxide anion (O_2_^−^) levels evaluated by dihydroethidium (DHE) fluorescence assay in bladders sections from control (saline) and cyclophosphamide (CYP, 300 mg/Kg, ip, 24 h)-injected animals untreated or treated (3 doses in 24 h) with the NOX1/4 inhibitor GKT137831 (5 mg/Kg, ip) or NOX2 inhibitor GSK2795039 (5 mg/Kg, ip). Fluorescence intensity, quantified by using ImageJ, and representative images from each group are shown in (**A**,**B**), respectively. Summary data (mean ± SEM) represent *n* = 6 for all groups. Significance was tested by two-way ANOVA followed by multiple comparisons by Tukey test. ∗*p* < 0.05 vs. CONTROL; #*p* < 0.05 vs. CYP.

In relation to the control samples, 24 h CYP exposure increased O_2_^−^ generation (DHE fluorescence intensity) by about of 55% in both smooth muscle and urothelial layers, as expected (Figure 7A,B). GKT137831 treatment did not dampen the CYP-induced O_2_^−^ production (*p* < 0.05 vs. the control), but GSK2795039 treatment significantly reduced the O_2_^−^ production by ~56% (*p* < 0.05 vs. CYP) although it remained above the control levels (*p* < 0.05). These results indicate that NOX2, rather than NOX1 or NOX4, is the predominant source of O_2_^−^ production in this model.

## 4. Discussion

Controlled generation of ROS results essentially from oxidases, which generate O_2_^−^ by donating an electron to O_2_, and whose concentration is dependent on the activity levels of SOD. Excessive amounts of ROS production or disruption of antioxidative pathways leads to oxidative stress, a key component of the underlying pathology of many diseases [18]. Mechanisms of oxidative damage and oxidative markers have been suggested as potent biomarkers for the IC/BPS disease diagnosis [12,34]. Preclinical studies [5,6,7,8,9,10,11] have implicated ROS in the pathogenesis of this disease, albeit the particular oxidative species and source(s) remains uncertain. In CYP-injected mice, our data showed that O_2_^−^ generation increased as early as 6 h after injection and remained high for up to 24 h, which was accompanied by reduced SOD activity, while H_2_O_2_ levels did not change. A typical response to increased O_2_^−^ is an increase in antioxidant defenses; however, SOD activity was significantly reduced 24 h after CYP-injection. Therefore, insufficient removal of excessive generated O_2_^−^ may be an additional mechanism of increased ROS in this model. Although many other enzymes can generate ROS, enzymatic systems predominate as the sources for ROS production, including NOX and uncoupled NO synthase. Under pathological conditions, due to enhanced oxidative stress, depletion of co-factors such as tetrahydrobiopterin, the eNOS may become dysfunctional resulting in production of large amounts of O_2_^−^ which may be produced by uncoupled eNOS [35]; however, eNOS was not found uncoupled structurally, as the ratio of dimers:monomers was unchanged after CYP exposure, or functionally, as inhibition of NOS by L-NAME did not reduce ROS generation. Thus, it is unlikely that eNOS is a source of the excessive O_2_^−^ observed in this model and, therefore, is unlikely to be the source of the excessive O_2_^−^ observed. Of all the ROS-generating enzymes, NOXs are of particular importance. NOX2 is known to be highly expressed in phagocytes [19]. NOX4 is constitutively active, contributing to basal ROS production, and it predominantly generates H2O2 rather than O2- [19]. NOX4 is highly expressed in the kidneys, where it was originally described [36]. However, our current knowledge about the role of NOXs in bladder physiology and disease is still poor. Few studies have explored the expression of these enzymes in urinary bladder samples. NOX4 is expressed at very low levels in human normal urothelium [24] whereas NOX1, NOX2, and NOX4 are overexpressed in urothelial carcinoma patients [24,37,38]. Here, we have demonstrated that NOX2 and NOX4 mRNAs were up- and downregulated, respectively, in the bladders of CYP-injected mice over time, while NOX1 remained unchanged. Whether elevated NOXs expression and NOX-derived ROS directly drive inflammation in CYP-induced cystitis is yet to be elucidated.

Studies with several NOX inhibitors have demonstrated that inhibition of NOX activity or expression may be of therapeutic value during pathological conditions [39]. Our results showed that selective inhibition of NOX2 by GSK2795039 improves CYP-induced bladder dysfunction and decreases bladder hyperactivity, inflammation, and vesical pain, whereas the dual NOX1/4 inhibitor GKT137831 had no significant protective effects.

GKT137831 was first reported as a dual NOX1/NOX4 inhibitor, with Ki-values in the nanomolar range for NOX1 and NOX4 and in the micromolar range for NOX2 [40]. In mice, GKT137831 ameliorated hypertensive cardiac remodeling [41], doxorubicin-induced cardiotoxicity [42], acute lung injury [27], acute kidney injury [43], and type I diabetes vascular dysfunction [44], the effects of which were attributed to inhibition of NOX1/4-driven ROS production in vivo. However, previous studies have demonstrated that GKT137831 interferes with peroxidase-dependent assays and has potent peroxynitrite [45] and hydrogen peroxide [46] scavenging activities.

GSK2795039 was the first identified selective NOX2 inhibitor [26]. Other compounds such as GLX481304 [47] and CYR5099 [48] were also recently discovered; however, GSK2795039 remains the reference NOX2 inhibitor. GSK2795039 was effective in abolishing the production of NOX2-derived ROS in vitro in cell-free and HL60 cells [26]. In addition, in mouse models of inflammation in the hind paw and in acute pancreatitis [26], as well as in traumatic brain injury [48], treatment with GSK2795039 exhibited the same protective effect as NOX2 deletion.

In freely moving mice, CYP exposure induced a pattern of voiding dysfunction, characterized by an increased number of urinary spots concomitant with smaller urinary volumes, which agrees with previous studies [5,6]. Additionally, in anaesthetized mice under CYP exposure, the filling cystometry revealed increases in micturition frequency and threshold pressure, accompanied by marked reductions in bladder capacity and compliance. Most of the micturition alterations in conscious and anaesthetized mice were significantly attenuated by GSK2795039, supporting the key role of NOX2 in voiding dysfunction in cystitis.

Furthermore, histological examination of urinary bladders showed that CYP exposure induces an increased bladder/body weight ratio, severe edema, especially at the submucosa, detrusor smooth muscle disorganization, and extensive urothelial denudation. In GSK2795039-treated animals exposed to CYP, minor structural changes of the bladder wall were observed and the urothelium was virtually intact, which is consistent with the reduced bladder/body weight ratio. Disruption of bladder barrier function integrity has been linked to the expression of antiproliferative factor [49], which may slow urothelial healing, favoring urinary diseases [50], including IC/BPS [51]. Damage to tight junction proteins is also implicated in the loss of bladder barrier integrity. Lower levels of occludin have been observed in both CYP-injected animals [7] and human IC/BPS [52]. GSK2795039 preserved the occludin mRNA of CYP-injected animals, suggesting that ROS-mediated cellular processes may be important during normal repair responses and that there is a potential regulatory role for NOX2 in bladder barrier integrity. Additional mechanisms may contribute to the protective effects of GSK2795039 on CYP-induced cystitis; for example, a direct effect on infiltrating inflammatory cells in which NOX2 is significantly expressed. In our study, treatment with GSK2795039 also greatly prevented vesical pain, which is a major feature of CYP-induced cystitis [4]. A previous study showed that mechanical and thermal hypersensitivity were attenuated in NOX2^-/-^ mice after peripheral nerve injury [53].

There are also some limitations to our study: (i) it is acknowledged that the CYP cystitis model is an animal model more relevant to the ulcerative form of IC/BPS; however, it is one of the most widely used and best characterized models to study this condition, and (ii) we did not evaluate the protein expression of NOXs, since commercially available antibodies often lack isoform specificity [54].

## 5. Conclusions

In summary, we have demonstrated the critical role of NOX2 in CYP-induced cystitis. Selective inhibition of NOX2 remarkably improved CYP-induced bladder damage, dysfunctional voiding behavior, and the vesical pain, a primary characteristic of cystitis. NOX2 inhibition emerges as a potential therapeutic target for IC/BPS and CYP chemotherapy-induced hemorrhagic cystitis.

## Figures and Tables

**Table 1:** Primer sequences (5′ → 3′) used for real-time PCR amplifications.

Gene	Forward	Reverse
*Nox1*	AATGCCCAGGATCGAGGT	GATGGAAGCAAAGGGAGTGA
*Nox2/Cybb*	TTGGGTCAGCACTGGCTCTG	TGGCGGTGTGCAGTGCTATC
*Nox4*	TGAACTACAGTGAAGATTTCCTTGAAC	GACACCCGTCAGACCAGGAA
*Ocln*	AAGTCAACACCTCTGGTGCC	TCATAGTGGTCAGGGTCCGT
*Actb*	GCAATGAGCGGTTCCGAT	TAGTTTCATGGATGCCACAGGAT
*18S rRNA*	GTAACCCGTTGAACCCCATT	CCAT CCAATCGGTAGTAGCG

Abbreviations: Actb, actin beta; Nox, NADPH oxidase isoform; Cybb, cytochrome B-245 beta Chain; Ocln, occludin; 18S rRNA, 18S ribosomal RNA.

## Data Availability

The data underlying this article will be shared on reasonable request to the corresponding author.

## References

[1] Doggweiler R., Whitmore K.E., Meijlink J.M., Drake M.J., Frawley H.C., Nordling J., Hanno P., Fraser M.O., Homma Y., Garrido G. (2016). A standard for terminology in chronic pelvic pain syndromes: A report from the chronic pelvic pain working group of the international continence society. Neurourol. Urodynamics.

[2] Dobberfuhl A.D. (2022). Pathophysiology, assessment, and treatment of overactive bladder symptoms in patients with interstitial cystitis/bladder pain syndrome. Neurourol. Urodyn..

[3] Emadi A., Jones R.J., Brodsky R.A. (2009). Cyclophosphamide and cancer: Golden anniversary. Nat. Rev. Clin. Oncol..

[4] Augé C., Chene G., Dubourdeau M., Desoubzdanne D., Corman B., Palea S., Lluel P., Vergnolle N., Coelho A.-M. (2013). Relevance of the cyclophosphamide-induced cystitis model for pharmacological studies targeting inflammation and pain of the bladder. Eur. J. Pharmacol..

[5] De Oliveira M.G., Calmasini F.B., Alexandre E.C., De Nucci G., Mónica F.Z., Antunes E. (2016). Activation of soluble guanylyl cyclase by BAY 58-2667 improves bladder function in cyclophosphamide-induced cystitis in mice. Am. J. Physiol. Physiol..

[6] De Oliveira M.G., Mónica F.Z., Calmasini F.B., Alexandre E.C., Tavares E.B.G., Soares A.G., Costa S.K.P., Antunes E. (2018). Deletion or pharmacological blockade of TLR4 confers protection against cyclophosphamide-induced mouse cystitis. Am. J. Physiol. Physiol..

[7] D’Amico R., Salinaro A.T., Cordaro M., Fusco R., Impellizzeri D., Interdonato L., Scuto M., Ontario M., Crea R., Siracusa R. (2021). Hidrox^®^ and Chronic Cystitis: Biochemical Evaluation of Inflammation, Oxidative Stress, and Pain. Antioxidants.

[8] Gonzalez E.J., Peterson A., Malley S., Daniel M., Lambert D., Kosofsky M., Vizzard M.A. (2015). The Effects of Tempol on Cyclophosphamide-Induced Oxidative Stress in Rat Micturition Reflexes. Sci. World J..

[9] Barut E.N., Engin S., Barut B., Kaya C., Kerimoglu G., Ozel A., Kadioglu M. (2019). Uroprotective effect of ambroxol in cyclophosphamide-induced cystitis in mice. Int. Urol. Nephrol..

[10] Liu Q., Wu Z., Liu Y., Chen L., Zhao H., Guo H., Zhu K., Wang W., Chen S., Zhou N. (2019). Cannabinoid receptor 2 activation decreases severity of cyclophosphamide-induced cystitis via regulating autophagy. Neurourol. Urodynamics.

[11] Ni B., Chen Z., Shu L., Shao Y., Huang Y., Tamrat N.E., Wei Z., Shen B. (2021). Nrf2 Pathway Ameliorates Bladder Dysfunction in Cyclophosphamide-Induced Cystitis via Suppression of Oxidative Stress. Oxidative Med. Cell. Longev..

[12] Jiang Y.-H., Jhang J.-F., Ho H.-C., Chiou D.-Y., Kuo H.-C. (2022). Urine Oxidative Stress Biomarkers as Novel Biomarkers in Interstitial Cystitis/Bladder Pain Syndrome. Biomedicines.

[13] Keleş I., Bozkurt M.F., Cemek M., Karalar M., Hazini A., Alpdağtaş S., Keleş H., Yildiz T., Ceylan C., Büyükokuroğlu M.E. (2014). Prevention of cyclophosphamide-induced hemorrhagic cystitis by resveratrol: A comparative experimental study with mesna. Int. Urol. Nephrol..

[14] Amanat S., Shal B., Seo E.K., Ali H., Khan S. (2022). Icariin attenuates cyclophosphamide-induced cystitis via down-regulation of NF-кB and up-regulation of Nrf-2/HO-1 signaling pathways in mice model. Int. Immunopharmacol..

[15] Katske F., Shoskes D.A., Sender M., Poliakin R., Gagliano K., Rajfer J. (2001). Treatment of interstitial cystitis with a quercetin supple-ment. Tech. Urol..

[16] Murina F., Graziottin A., Felice R., Gambini D. (2017). Alpha Lipoic Acid Plus Omega-3 Fatty Acids for Vestibulodynia Associated with Painful Bladder Syndrome. J. Obstet. Gynaecol. Can..

[17] Milkovic L., Cipak Gasparovic A., Cindric M., Mouthuy P.-A., Zarkovic N. (2019). Short Overview of ROS as Cell Function Regulators and Their Implications in Therapy Concepts. Cells.

[18] Casas A.I., Nogales C., Mucke H.A.M., Petraina A., Cuadrado A., Rojo A.I., Ghezzi P., Jaquet V., Augsburger F., Dufrasne F. (2020). On the Clinical Pharmacology of Reactive Oxygen Species. Pharmacol. Rev..

[19] Vermot A., Petit-Härtlein I., Smith S., Fieschi F. (2021). NADPH Oxidases (NOX): An Overview from Discovery, Molecular Mechanisms to Physiology and Pathology. Antioxidants.

[20] Ogboo B.C., Grabovyy U.V., Maini A., Scouten S., van der Vliet A., Mattevi A., Heppner D.E. (2022). Architecture of the NADPH oxidase family of enzymes. Redox Biol..

[21] Calmasini F.B., de Oliveira M.G., Alexandre E.C., Silva F.H., Tavares E.B., André D.M., Zapparoli A., Antunes E. (2017). Obesity-induced mouse benign prostatic hyperplasia (BPH) is improved by treatment with resveratrol: Implication of oxidative stress, insulin sensitivity and neuronal growth factor. J. Nutr. Biochem..

[22] Alexandre E.C., Calmasini F.B., Sponton A.C.S., de Oliveira M.G., André D.M., Silva F., Delbin M., Mónica F.Z., Antunes E. (2018). Influence of the periprostatic adipose tissue in obesity-associated mouse urethral dysfunction and oxidative stress: Effect of resveratrol treatment. Eur. J. Pharmacol..

[23] Huang H.-S., Liu Z.-M., Chen P.-C., Tseng H.-Y., Yeh B.-W. (2012). TG-interacting factor-induced superoxide production from NADPH oxidase contributes to the migration/invasion of urothelial carcinoma. Free Radic. Biol. Med..

[24] Shimada K., Fujii T., Anai S., Fujimoto K., Konishi N. (2011). ROS generation via NOX4 and its utility in the cytological diagnosis of urothelial carcinoma of the urinary bladder. BMC Urol..

[25] Elbatreek M.H., Mucke H., Schmidt H.H.H.W. (2020). NOX Inhibitors: From Bench to Naxibs to Bedside. Reactive Oxygen Species.

[26] Hirano K., Chen W.S., Chueng A.L., Dunne A.A., Seredenina T., Filippova A., Ramachandran S., Bridges A., Chaudry L., Pettman G. (2015). Discovery of GSK2795039, a Novel Small Molecule NADPH Oxidase 2 Inhibitor. Antioxidants Redox Signal..

[27] Cui Y., Wang Y., Li G., Ma W., Zhou X.-S., Wang J., Liu B. (2018). The Nox1/Nox4 inhibitor attenuates acute lung injury induced by ischemia-reperfusion in mice. PLoS ONE.

[28] Gorin Y., Cavaglieri R.C., Khazim K., Lee D.-Y., Bruno F., Thakur S., Fanti P., Szyndralewiez C., Barnes J.L., Block K. (2015). Targeting NADPH oxidase with a novel dual Nox1/Nox4 inhibitor attenuates renal pathology in type 1 diabetes. Am. J. Physiol. Physiol..

[29] Davel A.P., Victorio J.A., Delbin M.A., Fukuda L.E., Rossoni L.V. (2015). Enhanced endothelium-dependent relaxation of rat pulmonary artery following β-adrenergic overstimulation: Involvement of the NO/cGMP/VASP pathway. Life Sci..

[30] Oliveira A.L., Medeiros M.L., de Oliveira M.G., Teixeira C.J., Mónica F.Z., Antunes E. (2022). Enhanced RAGE Expression and Excess Reactive-Oxygen Species Production Mediates Rho Kinase-Dependent Detrusor Overactivity After Methylglyoxal Exposure. Front. Physiol..

[31] Kim A.K., Hamadani C., Zeidel M.L., Hill W.G. (2020). Urological complications of obesity and diabetes in males and females of three mouse models: Temporal manifestations. Am. J. Physiol. Physiol..

[32] Yu W., MacIver B., Zhang L., Bien E.M., Ahmed N., Chen H., Hanif S.Z., de Oliveira M.G., Zeidel M.L., Hill W.G. (2022). Deletion of mechanosensory β1-integrin from bladder smooth muscle results in voiding dysfunction and tissue remodeling. Function.

[33] Deuis J.R., Dvorakova L.S., Vetter I. (2017). Methods Used to Evaluate Pain Behaviors in Rodents. Front. Mol. Neurosci..

[34] Ener K., Keske M., Aldemir M., Ozcan M.F., Okulu E., Özayar A., Ergin M., Doluoglu O.G., Çakmak S., Erel O. (2015). Evaluation of oxidative stress status and antioxidant capacity in patients with painful bladder syndrome/interstitial cystitis: Preliminary results of a randomised study. Int. Urol. Nephrol..

[35] Łuczak A., Madej M., Kasprzyk A., Doroszko A. (2020). Role of the eNOS Uncoupling and the Nitric Oxide Metabolic Pathway in the Pathogenesis of Autoimmune Rheumatic Diseases. Oxidative Med. Cell. Longev..

[36] Geiszt M., Kopp J.B., Várnai P., Leto T.L. (2000). Identification of Renox, an NAD(P)H oxidase in kidney. Proc. Natl. Acad. Sci. USA.

[37] Shimada K., Nakamura M., Anai S., De Velasco M., Tanaka M., Tsujikawa K., Ouji Y., Konishi N. (2009). A Novel Human AlkB Homologue, ALKBH8, Contributes to Human Bladder Cancer Progression. Cancer Res..

[38] Shimada K., Fujii T., Tsujikawa K., Anai S., Fujimoto K., Konishi N. (2012). ALKBH3 Contributes to Survival and Angiogenesis of Human Urothelial Carcinoma Cells through NADPH Oxidase and Tweak/Fn14/VEGF Signals. Clin. Cancer Res..

[39] Chocry M., Leloup L. (2020). The NADPH Oxidase Family and Its Inhibitors. Antioxidants Redox Signal..

[40] Teixeira G., Szyndralewiez C., Molango S., Carnesecchi S., Heitz F., Wiesel P., Wood J.M. (2016). Therapeutic potential of NADPH oxidase 1/4 inhibitors. Br. J. Pharmacol..

[41] Zeng S.-Y., Yang L., Yan Q.-J., Gao L., Lu H.-Q., Yan P.-K. (2018). Nox1/4 dual inhibitor GKT137831 attenuates hypertensive cardiac remodelling associating with the inhibition of ADAM17-dependent proinflammatory cytokines-induced signalling pathways in the rats with abdominal artery constriction. Biomed. Pharmacother..

[42] Zheng H., Xu N., Zhang Z., Wang F., Xiao J., Ji X. (2022). Setanaxib (GKT137831) Ameliorates Doxorubicin-Induced Cardiotoxicity by Inhibiting the NOX1/NOX4/Reactive Oxygen Species/MAPK Pathway. Front. Pharmacol..

[43] Jeong B.Y., Lee H.Y., Park C.G., Kang J., Yu S.-L., Choi D.-R., Han S.-Y., Park M.H., Cho S., Lee S.Y. (2018). Oxidative stress caused by activation of NADPH oxidase 4 promotes contrast-induced acute kidney injury. PLoS ONE.

[44] Gray S.P., Jha J.C., Kennedy K., van Bommel E., Chew P., Szyndralewiez C., Touyz R.M., Schmidt H.H.H.W., Cooper M.E., Jandeleit-Dahm K.A.M. (2017). Combined NOX1/4 inhibition with GKT137831 in mice provides dose-dependent reno- and atheroprotection even in established micro- and macrovascular disease. Diabetologia.

[45] Sedeek M., Gutsol A., Montezano A.C., Burger D., Cat A.N.D., Kennedy C.R.J., Burns K.D., Cooper M.E., Jandeleit-Dahm K., Page P. (2012). Renoprotective effects of a novel Nox1/4 inhibitor in a mouse model of Type 2 diabetes. Clin. Sci..

[46] Schildknecht S., Weber A., Gerding H.R., Pape R., Robotta M., Drescher M., Marquardt A., Daiber A., Ferger B., Leist M. (2013). The NOX1/4 Inhibitor GKT136901 as Selective and Direct Scavenger of Peroxynitrite. Curr. Med. Chem..

[47] Szekeres F.L.M., Walum E., Wikström P., Arner A. (2021). A small molecule inhibitor of Nox2 and Nox4 improves contractile function after ischemia–reperfusion in the mouse heart. Sci. Rep..

[48] Wang M., Luo L. (2020). An Effective NADPH Oxidase 2 Inhibitor Provides Neuroprotection and Improves Functional Outcomes in Animal Model of Traumatic Brain Injury. Neurochem. Res..

[49] Keay S., Leitzell S., Ochrzcin A., Clements G., Zhan M., Johnson D. (2012). A mouse model for interstitial cystitis/painful bladder syndrome based on APF inhibition of bladder epithelial repair: A pilot study. BMC Urol..

[50] Kreft M.E., Hudoklin S., Jezernik K., Romih R. (2010). Formation and maintenance of blood–urine barrier in urothelium. Protoplasma.

[51] Zhang C.-O., Wang J.-Y., Koch K.R., Keay S. (2005). Regulation of tight junction proteins and bladder epithelial paracellular permeability by an antiproliferative factor from patients with interstitial cystitis. J. Urol..

[52] Lee J.-D., Lee M.-H. (2014). Decreased expression of zonula occludens-1 and occludin in the bladder urothelium of patients with interstitial cystitis/painful bladder syndrome. J. Formos. Med. Assoc..

[53] Kim D., You B., Jo E.-K., Han S.-K., Simon M.I., Lee S.J. (2010). NADPH oxidase 2-derived reactive oxygen species in spinal cord microglia contribute to peripheral nerve injury-induced neuropathic pain. Proc. Natl. Acad. Sci. USA.

[54] Diebold B.A., Wilder S.G., De Deken X., Meitzler J.L., Doroshow J.H., McCoy J.W., Zhu Y., Lambeth J.D. (2019). Guidelines for the Detection of NADPH Oxidases by Immunoblot and RT-qPCR. NADPH Oxidases.

